# 

**Published:** 1888-11-10

**Authors:** 


**Notice to Advertisers and Subscribers.**

All Advertisements, Orders for Copies of The Hospital, and Business Communications, should be addressed to The Publisher, *not to the Editor*, The Hospital, 38, Old Jewry, E.C.

A Scale of Charges will be found in the Advertisement Sheets, page iii.

**To Residents, Secretaries, and Matrons.**

The Editor will be glad to have the Regulations and each Report as issued by Asylums, Hospitals, Convalescent and Nursing Institutions, as well as those of other Charities, and an early copy in duplicate of all Appeals and Festival Notices, etc., addressed to *The Editor, The Lodge, Porchester Square, London, W.* Any superintendent, secretary, matron, or other chief official who regularly sends such information may be registered, on application to the Publisher, to receive free of cost a cover for binding The Hospital in half-yearly volumes.

### THE STUDENT'S COLUMN.

### ROYAL COLLEGES OF PHYSICIANS AND SURGEONS, EDINBURGH, AND FACULTY OF PHYSICIANS AND SURGEONS, GLASGOW.

THE following are the questions which were given at the recent examinations for the Qualification in Medicine and Surgery conducted by the Royal Colleges of Physicians and Surgeons, Edinburgh, and the Faculty of Physicians and Surgeons, Glasgow:—

### FIRST EXAMINATION.

QUESTIONS IN CHEMISTRY (four questions, all of which are to be answered).—I. Name the usual instrument employed for the measurement of temperature, and give a brief description of its construction. II. Describe the preparation of nitric acid on the manufacturing scale, giving the equation. State its more important properties, and the tests for it when free and combined. III What is "white arsenic"? State how it is obtained. Describe Reinsch's test for arsenic. IV. How is ether prepared? Give the equations, and mention its chief properties.

QUESTIONS IN ELEMENTARY ANATOMY (all of which are to be answered).—I. Enumerate the bones which are concerned in the formation of the orbit, and mention their relative position. II. Name the ligaments by which two typical vertebræ are connected together. III. Give the course, relations, and branches of the popliteal artery. IV. Describe the origin, course, mode of termination, and relations of the musculo-spiral nerve.

### SECOND EXAMINATION.

QUESTIONS IN MATERIA MEDICA (three questions, all of which are to be answered).—I. Mention three diuretics, adding the appropriate doses. II. Mention three antipyretic drugs and their doses. III. How is the vinum ipecacuanhæ prepared? Give the composition of the pilula ipecacuanhæ cum scilla.

QUESTIONS IN ADVANCED ANATOMY (all of which are to be answered).—I. Give the origin, course, relations, and distribution of the phrenic nerve. II. Name the branches given off by the external carotid artery, and describe those derived from its posterior aspect. III. Describe the external oblique muscle of the abdomen. IV. What is the usual arrangement of the peritoneum where it covers the cæcum? Describe the cæcum and iliocæcal valve.

QUESTIONS IN PHYSIOLOGY (all of which are to be answered).—I. Describe how the first blood capillaries and blood corpuscles are formed in the fœtus. II. Describe the act of deglutition, and state what muscles are concerned, what their nerve supply is, where the nerve centre that governs the act is placed, and what are the afferent nerves. III. What are the causes of the cardiac sounds? IV. Describe the function of the organ of corti, and of the semicircular canals.

### FINAL EXAMINATION.

QUESTIONS IN MEDICINE (three only of which are to be answered).—I. Enumerate and explain the initial symptoms of locomotor ataxia, and give the anatomical changes in the spinal cord in the first stage of the disease. II. Define purpura. Describe the varieties of the disease. Give the diagnosis from scorbutus, and the treatment. III. Give the symptoms and pathology of gastric ulcer. State fully, and explain the reasons for the treatment you would employ in this disease. IV. Give the diagnosis, morbid anatomy, and treatment of favus.

QUESTIONS IN THERAPEUTICS (all of which are to be answered, including the prescription).—I How does iodide of potassium prove of service in aneurism? Detail the form of diet to be observed in such a case. II. How may an acute attack of angina pectoris sometimes be speedily relieved? and what means should be adopted to prevent a recurrence of the attack? III. By what external applications may neuralgia be relieved? and how do the remedies usually employed internally bring about a cure? Prescription (to be written in unabbreviated Latin, the directions in English).—Prescribe acid tartrate of potash in the form of electuary.

QUESTIONS IN SURGERY (all of which are to be answered).—I. Caries of the bodies of the vertebræ—(1) What are the causes of death in this disease? (2) Where do the abscesses connected with cervical, dorsal, and lumbar caries usually discharge themselves. II. Popliteal aneurism—Describe the methods of treatment by flexion, by arterial compression, and by ligature. III. What structures are apt to be ruptured in subcoracoid dislocation of the humerus? Give the symptoms of that dislocation. IV. Describe excision of the upper jaw.

SURGICAL ANATOMY (one of which only is to be answered).—I. Give the distribution of the external popliteal nerve, and the effects produced by its division. II. What is the course of the lingual artery? Mention in order from without inwards the structures seen in cutting down upon it.

QUESTIONS IN MEDICAL JURISPRUDENCE AND HYGIENE (four questions, of which three are to be answered, and in these, three and four must be included).—I. Mention some diseases which are apt to be feigned, and state how you would proceed to investigate them. II. What are the symptoms produced by starvation, when food and drink are both unattainable? How long does life generally last in such circumstances? and describe the appearances presented shortly before death, as also those found *post mortem*. III. What is opium? Mention its two most prominent active principles What is the strength, and what would be a poisonous dose of tincture of opium for an adult, the symptoms produced by it, and with what diseases might such a case be confounded? giving the diagnostic points of each. How would you treat a case of opium poisoning? IV. Describe the A B C method of the disposal of sewage, and state any advantages or disadvantages possessed by it.

### Scraps and Gleanings.

Two Hundred pounds has been spent on disinfecting a thousand houses after the sewage floods in the Isle of Dogs.

There are now two patients in the Lodge Moor Hospital, so it seems that Sheffield is not yet free from small-pox.

A bust of the late Dr. Wilson Fox, from the chisel of Miss Margaret Thomas, has been unveiled at the Shirehall, Taunton.

The *Northampton Mercury* is again offering prizes for home-made toys, which will be distributed amongst the small inmates of the neighbouring hospitals and workhouses at Christmas.

Pads of spagnum moss are being tested as compresses for wounds in several London hospitals.

The report just issued by the Eye Infirmary, Exeter, tells of five hundred patients cured and a thousand benefited during the year.

The following are the latest moves amongst naval surgeons: Staff Surgeons J. A. Robertson, to the *Penelope*; R. D. White, to the *Hotspur*; M. O C. M'Swiney to the Haslar Hospital, temporary; Surgeon J. W. Slaughter to the *Pheasant*.

The addition to the Birmingham Orthopædic Hospital is expected to be complete by Christmas; but unless further subscribers come forward extra patients cannot be admitted.

On Hospital Sunday at Birmingham £2,336 were received at the office of the fund, against £2,743 received on the evening of Hospital Sunday last year.

Concerts are being given at Harrow, under the direction of Colonel Cleather, in aid of the Cottage Hospital.

The new children's ward to the Hereford Infirmary will be opened about three weeks hence.

Canon Tinling preached an excellent sermon in Gloucester Cathedral on October 21st in aid of the medical charities of the town. The collection amounted to over £15.

A three days bazaar in aid of the hospital, was held at the Boston Assembly Rooms last week. It was hoped to realise £200 to clear the debt on he hospital, but the result was far below the expectations.

Mrs. Reaney is trying to start a convalescent and holiday home at Blackpool, similar to the one she started at Folkestone five years ago.

The necessary three thousand pounds for the building of a children's hospital at Belfast has been raised.

Many women feel it is a great pity that the Lambeth Guardians will not admit Dr. Annie McCall's students to the infirmary wards.

It is proposed to abolish the army medical school connected with Netley Hospital.

The erection of a fourth lunatic asylum for Middlesex has been sanctioned by the magistrates. The cost will be about £22,000.

Dr. J. G. Gardener has been appointed house-surgeon and secretary of Stourbridge Dispensary, in place of Dr. Daly, resigned.

Plans are now being considered for the erection of a new Cottage Hospital at Longton, on the site presented by the Duke of Sutherland.

A committee has been appointed to inquire into the management of the Staffordshire General Infirmary.

Hospital Sunday at Salisbury has resulted in £203, as compared with £201 collected last year.

At the quarterly meeting of Governors of Radcliffe Infirmary, Oxford, on October 24th there was a rather warm discussion about the proposal to raise the medical officers' salaries. Finally the vexed question was postponed till the January court.

The Bishop of Peterborough preached a special sermon at Northampton on October 21st to the Oddfellows. The collection, which was given to the Infirmary, amounted to £40.

It is proposed to build a Foundling Hospital at Melbourne, Victoria. There are many who object to the scheme.

The *County Government Review* describes the new hospital at Walker, near Newcastle, as a model infectious hospital.

Dr Morrison has been elected assistant surgeon of Newcastle Infirmary, in place of Dr. Williamson resigned.

It is hoped that the Convalescent Home for Friendly Society Members, Durham will be built by May 1st, 1889.

Forres has secured the necessary £2,000 for its cottage hospital.

Mrs. Eyre Crabbe has left £200 to the Royal South Hants Infirmary, with which she was long connected. At the meeting of the governors last week, a motion was passed expressing sympathy and condolence with Major Eyre Crabbe.

The total amount collected at Whitstable in connexion with the Hospital Saturday and Sunday Funds was £62, of which £22 was collected in the workshops.

The St. John's Ambulance Association flourishes in Australia, having over 100 centres.

The treasurer of Leicester Infirmary acknowledges the receipt of £877 from the Hospital Sunday collections.

During the last year the Manchester Southern Hospital has had 64 maternity cases in the hospital, and the midwives have attended nearly double the usual number of outdoor cases.

The children of Cardiff held a bazaar last week in the interests of the Eye and Ear Hospital of that town.

The late house-to-house collection for Lincoln Hospital secured that institution £600.

The Mayor of Liverpool has given £25 towards £500 wanted immediately to build an annexe to St. Paul's Eye and Ear Hospital.

The new infectious hospital at Salford will cost close upon £40,000. Some of the ratepayers are grumbling at the largeness of the sum.

Mr. W. O'Hanlon has been denouncing the system of recommendations in vogue at Ancoats Hospital Manchester, in the local press.

Mrs. Temple, wife of the Bishop of London, makes an appeal on behalf of the Inebriate Home for Women, under the auspices of the Church of England Women's Union Temperance Society.

### Amusements and Relaxation.

### NEW PUZZLE PRIZE SCHEME.

**COMMENCED OCTOBER 6th, ENDS DECEMBER 29th, 1888.**

On October the 6th a New Prize Scheme was commenced in these columns. Prizes will be awarded for the best Original Puzzles in Prose or Verse, relating to any of the Social, Philanthropic, or Political topics of the day, and taking the form of Double Acrostics, Anagrams, Square Words, Decapitations, Transpositions, or any other Puzzles which the ingenuity of the competitors may suggest. (*Answers must accompany contributions.*)

*Readers* are requested to send in weekly the Number of Marks they consider each composition to be entitled to, three being the highest score for any one contribution.

Eight Prizes of Standard Books will be given, the choice of the book to be left to the winner.

First, Second, Third, Fourth, and Fifth Prizes for best Original Puzzles, of £1 1s., 15s., 10s. 6d., 7s. 6d., and 5s. each.

First, Second, and Third Prize for Solutions of 15s., 10s. 6d., and 5s. each.

N.B.—All contributions must reach the office, 38, Old Jewry, E.C., not later than the *First Post* on *Thursday* in each week.

### N.B.—SPECIAL NOTICE.

We have this week received a variety of puzzles in prose and verse, all showing more or less ingenuity in their production, but by the non-observance of the rule heading this column, that all *puzzles must relate to some of the social, political, or philanthropic topics of the day*, many competitors are debarred from receiving the number of marks to which they otherwise would be entitled. A "Topical Alphabet" embracing all the three given subjects would be an amusing and interesting composition. Who will try his hand at it? It is better to forward *one*, or *even two*, really good puzzles each week than several indifferent ones.

### PUZZLES FOR SOLUTION.

**Fourth Week.**

No. 10. Double Acrostic.

Stiff will the contest be, mid fog and mist,
The smaller number hasten to the Gray;
The men of science on their side enlist,
And shed true light on autumn's dreary day.

1. A number sacred to the classic groves.
2. This marked the consecration of a king.
3. In this the youthful babble of their loves.
4. By this transmitted, swiftly our thought takes wing.
5. Unworthy inmate of a noble brain.
6. Friend of a Father warring with a son.
7. From neighbouring height she cheers the silent plain.
8. Bless us, O Goddess, till our race be run. "A. M. E."

No. 11. Decapitation.

Many think that I should bring
Prosperity upon my wing.
Cut off my head, and you will see
What must be done, if you try me.
Cut off a third of what remains
You'll have refreshment for your pains. "Tinie."

No. 12. Charade.

My *first* means equality.
My *second* falls with solemn cadence on the ear,
My *whole* is a man whose aim is very clear.
Severing our unity. "Gretta."

**Marks.**

| Names. | No. of Puzzles sent in Nov. 1. | No. of Marks Nov. 1 | Total of Marks. | Names. | No. of Puzzles sent in Nov. 1. | No. of Marks Nov. 1. | Total of Marks. |
|---|---|---|---|---|---|---|---|
| Patience .... | 3 | 5 | 22 | Hercules .. | 6 | 0 | 9 |
| Lady Clara.. | 2 | 5 | 24 | Gretta ...... | 2 | 4 | 12 |
| Tinie ...... | 3 | 6 | 17 | Dandy Dick | — | — | 5 |
| Bijou ...... | 1 | 0 | 8 | Boadicea .. | — | — | 7 |
| G. D. ...... | 2 | 3 | 6 | Nipper .... | 5 | 5 | 12 |
| Thanet .... | 2 | 2 | 16 | Morico .... | 2 | 4 | 4 |
| Daisy ..... | 2 | 2 | 8 | A. M. E..... | 1 | 3 | 3 |
| Salford .... | — | — | 6 | | | | |

**Answers.**

No. 4. Double Acrostic.

| | | |
|---|---|---|
| L | am | P |
| O | yste | R |
| R | ienz | I |
| D | ru | M |
| S | ilenc | E |
| A | laru | M |
| L | ev | I |
| I | nvasio | N |
| S | cutar | I |
| B | lunderbus | S |
| U | trech | T |
| R | omanc | E |
| Y | ea | R |

No. 5. Original Anagram.

The Mission to Deep Sea Fishermen.

No. 6. Multum in Parvo Puzzle.—Dishearten.—1, Dish; 2, Is; 3, Shear; 4, She; 5, He; 6, Heart; 7, Hear; 8, Ear; 9, Art; 10, Ten.

**Marks for Solution of Puzzles.**

| | Nov. 1. | | Total. | | Nov. 1. | | Total. |
|---|---|---|---|---|---|---|---|
| Ruffles.......... | 1 | .. | 3 | Marguerite.... | 2 | .. | 5 |
| Daisy .......... | 1 | .. | 3 | Patience ...... | 1 | .. | 2 |
| Lady Clara...... | 2 | .. | 4 | Gretta ........ | — | .. | 2 |
| Reldas.......... | 2 | .. | 4 | Amicus ...... | 1 | .. | 1 |
| Mater .......... | 1 | .. | 2 | Scrooge ...... | 2 | .. | 4 |
| Condorcet ...... | 1 | .. | 3 | Smut ........ | 2 | .. | 2 |
| Tinie .......... | 2 | ... | 3 | Thanet........ | 3 | .. | 6 |
| Gentian ........ | 2 | .. | 5 | | | | |

(*For Conditions, see last week's* The Hospital.)

